# Bacterial Interactions with Dental and Medical Materials

**DOI:** 10.3390/jfb11040083

**Published:** 2020-11-23

**Authors:** Mary Anne Melo

**Affiliations:** 1 Division of Operative Dentistry, Department of General Dentistry, University of Maryland School of Dentistry, 650 W. Baltimore St, Baltimore, MD 21201, USA; MMelo@umaryland.edu; 2 Dental Biomedical Science Ph.D. Program, University of Maryland School of Dentistry, 650 W. Baltimore St, Baltimore, MD 21201, USA

Fundamental scientific understanding of oral diseases associated with tissue-contacting dental and medical devices is primordial to facilitate pathways for their translation to clinical use. The interaction of bacteria with biomaterials’ surfaces has critical clinical implications due to biofilm formation and biofouling. Although biofilms play an important positive role in various ecosystems, they also have many adverse effects, including biofilm-related infections in medical and dental settings.

Biofilms account for up to 80% of the total number of bacteria-related infections, including endocarditis, cystic fibrosis, secondary dental caries, periodontitis, rhinosinusitis, osteomyelitis, non-healing chronic wounds, meningitis, kidney infections, and prosthesis- and implantable device-related infections.

Worldwide, populations of all ages suffer from oral biofilm-trigged diseases, such as dental caries and tooth surrounding tissue infections impacting the human body. New dental materials and biomaterials, currently being introduced, under development, or proposed, are expected to benefit the oral health status. These materials will provide a wide range of diverse functions, from promoting osteogenesis to bacterial biofilm formation inhibition. 

In the Special Issue “Bacterial Interactions with Dental and Medical Materials,” encouraging findings on tissue-contacting biomaterials to control biofilms and susceptibility to bacterial colonization and understand mechanisms, clinical perspectives beneficial to healthcare were discussed.

As cariogenic bacterial grows inside the mouth due to lack of caries disease management, the tooth–material interface continues to degrade by bacterial acids leading to increasing premature failure of tooth filling. Bienek et al. [1], Mena Silva et al. [2], Yaghmoor et al. [3], Lu and Jin [4], Lygidakis et al. [5], and Mitwalli et al. [6] investigated the application of polymerizable antibacterial monomers based on di-imidazolium or quaternary ammonium compounds as an antibacterial strategy for resin-based materials in efforts toward caries-related biofilm control.

Looking for the control of and reduction in oral biofilm formation, initiated by bacterial species living in polymicrobial, pathogenic colonies at or below the gingival margin, Mortazavian et al. [7], Afonso Camargo et al. [8], and Torres and Bienek [9] presented antibiofilm strategies targeting adhesion to a substrate via brush-like structures of highly packed polymer chains and silicon carbide coating that could physically repel bacteria to result in a significant reduction in attached bacteria. 

The search for new bioactive compounds to prevent dental caries development and progression has led researchers to focus their attention on the use of nanotechnologies, especially hydroxyapatite (HAp), metals, and metal oxide nanoparticles. Garcia et al. [10] investigate the effects of different loadings of cerium dioxide on their radiopacity and degree of conversion of dental adhesives. Nanotechnology applications have offered the opportunity to modulate the formation of dental biofilms using nanoparticles with bioactive effects. Luong et al. [11] have assessed novel nanohydroxyapatite-based desensitizer and its effect on dental adhesives’ bond strength.

Ionescu et al. [12] have looked to the synergistic antibacterial performance of toothpaste containing nano-hydroxyapatite substituted with metal ions. Shimabukuro et al. [13] have investigated both the durability of the antibacterial effect and the surface change of Ag- and Cu-incorporated porous titanium dioxide (TiO_2_) layer; silver (Ag) and copper (Cu) have been incorporated into the titanium (Ti) surface to realize their antibacterial properties. Ag- and Cu-incorporated TiO_2_ layers were formed by micro-arc oxidation (MAO) treatment using the electrolyte with Ag and Cu ions. Their collective findings indicated the importance of the time-transient effects of Ag and Cu. This knowledge will help design antibacterial implants based on Ag and Cu’s surface changes. Paterson et al. [14] incorporate silver-doped nano-hydroxyapatite into electrospun scaffolds for applications in bone repair.

Papynov et al. [15] have developed a combination of calcium monosilicate, β-wollastonite (CaSiO_3_), and hydroxyapatite (HAp), which is a complete analog of a living bone. This approach can pave the way to the fabrication of biocompatible ceramics for bone tissue engineering; thus, contributing another flexible strategy for the synthesis of biomaterials with broad intended applications in traumatology, orthopedics, dentistry, maxillofacial surgery, and other areas of medicine for the recovery, replacement, and reconstruction of the damaged tissue.

As we observe an increasing number of investigations addressing new antibacterial materials and devices, we also face a significant challenge in evaluating the new materials. There is a limited understanding of the mechanisms involved in bacteria–materials interactions in the oral environment. The gaps in the development and application of reliable methodological approaches for the characterization of this new generation of antibacterial materials require considering the complexity and heterogeneity of the disease process investigated.

Garcia-gareta et al. [16] developed a live ex vivo model of persistent infection that can be used for pre-screening biomaterials intended for treating chronic wounds for their antimicrobial and angiogenic potential. This model is relatively simple, quick, and low-cost and mimics the in vivo situation more closely than traditionally used antimicrobial tests using agar plates and dilution assays. Additionally, keeping under the principles of the National Centre for the Replacement Refinement and Reduction of Animals in Research, this model does not require administrative procedures for obtaining ethics committee approval for animal experimentation.

In this Special Issue, there are 16 papers by authors from 9 countries in Asia, Europe, and Latin and North America. Their investigations represent a wide range of aspects related to the current scenario on bacteria interactions with biomaterial surfaces and give timely examples of research activities that can be observed around the globe. We hope that this work will be inspirational for further research on understanding oral diseases associated with tissue-contacting dental and medical devices, new antibiofilm agents, and the relevance of assessing the material using clinically significant biofilm models.
